# Sex-specific association between elective cesarean section and growth trajectories in preschool children: A prospective birth cohort study

**DOI:** 10.3389/fpubh.2022.985851

**Published:** 2022-09-20

**Authors:** Shanshan Zhang, Jixing Zhou, Mengting Yang, Fu Zhang, Xingyong Tao, Fangbiao Tao, Kun Huang

**Affiliations:** ^1^Department of Maternal, Child & Adolescent Health, School of Public Health, Anhui Medical University, Key Laboratory of Population Health Across Life Cycle (Anhui Medical University), Ministry of Education of the People's Republic of China, NHC Key Laboratory of Study on Abnormal gametes and Reproductive Tract, Anhui Provincial Key Laboratory of Population Health and Aristogenics, Hefei, China; ^2^Scientific Research Center in Preventive Medicine, School of Public Health, Anhui Medical University, Hefei, China

**Keywords:** cesarean section, BMI trajectory, adiposity rebound, birth cohort, repeated anthropometric data

## Abstract

**Background:**

Elective cesarean section (ECS) primarily contributes to the rising cesarean section (CS) rate, and much attention has been attracted to its health consequences. The association between ECS and overweight and obesity in children has been controversial, and few studies distinguished ECS with medical indications from those without indications. Based on a large sample birth cohort, we aim to examine the association of ECS with or without medical indications on children's physical development by using repeated anthropometric data from birth to 6 years of age.

**Methods:**

A total of 2304 mother-child pairs with complete data on delivery mode and children's anthropometric measurements were recruited from the Ma'anshan-Anhui Birth Cohort (MABC) in China. ECS was the main exposure in this study, and the primary outcomes were children's growth trajectories and early adiposity rebound (AR). Children's BMI trajectories were fitted by using group-based trajectory models and fractional polynomial mixed-effects models. The association between ECS and children's growth trajectories and early AR was performed using multiple logistic regression models.

**Results:**

Among 2,304 mother-child pairs (1199 boys and 1105 girls), 1088 (47.2%) children were born by CS, including 61 (5.6%) emergency CS, 441 (40.5%) ECS with medical indications, and 586 (53.9%) ECS without medical indications. After adjusting for potential confounders, it was found that ECS with medical indications was associated with a “high level” of BMI trajectory (OR = 1.776; 95% CI: 1.010–3.123), and ECS without medical indications was associated with early AR (OR = 1.517; 95% CI: 1.123–2.050) in girls. In boys, we found that ECS without medical indications was unlikely to experience an accelerated growth trajectory (OR = 0.484; 95%CI: 0.244–0.959).

**Conclusions:**

ECS may be related to girls' “high level” BMI trajectories and early AR. If causal, the findings will provide an evidence-based reference for early life interventions for childhood obesity.

## Introduction

With the development of perinatal medicine, cesarean section (CS) has become an effective means of saving the lives of women and babies in the event of serious obstetric complications. Recent data (2010–2018) based on 154 countries covering 94.5% of live births worldwide shows that, on average, 21.1% of women have childbirth by CS. It is estimated that 28.5% of women worldwide will deliver by CS by 2030 (1). The domestic cesarean rate increased from 28.8% in 2008 to 36.7% in 2018 (2).

CS is divided into emergency CS and elective CS (ECS) according to the presence or absence of labor, and ECS is subdivided into ECS with medical indications and ECS without medical indications. Studies have shown that ECS without medical indication is the main contributor to the increasing CS rate (3).

At present, there is no evidence that CS without medical indications benefits the mother and the baby. Also, WHO states that CS rates above 10% are no longer associated with a reduction in maternal and infant mortality. Actually, CS carries risks and may have health consequences for women and children (4). Infants born *via* CS have different hormonal and microbial exposures from vaginal delivery, and that can subtly alter the physiology of the newborn (5). The health effects of CS on children have been shown to include altered immune development, increased risk of allergy and asthma, and reduced gut microbial diversity (5).

The increasing prevalence of childhood obesity has become a worldwide public health problem, with 39 million children under 5 years of age being overweight or obesity worldwide in 2020 (6). Obesity children would experience respiratory distress, increased risk of fracture, hypertension, early signs of cardiovascular disease, insulin tolerance and psychological problems. They are also at great risk of obesity, premature death and disability in adulthood (6).

The high rate of CS and the simultaneous high prevalence of obesity have led scholars to become interested in the effect of CS on offspring's overweight and obesity. Previous studies have looked closely at the association between CS and offspring's overweight and obesity, but the findings are controversial. Mulleer NT et al (7) found that CS was associated with higher rates of weight gain in the first year of life in offspring compared to vaginal delivery. Evidence from American GUTS cohort showed that cesarean delivery was associated with 15% increased risk of offspring's obesity (8). Chinese studies similarly revealed that CS was correlated with overweight and obesity in offspring (9, 10). However, a cohort study in Ireland showed no association between mode of delivery and obesity in children aged 2 and 5 years (11). However, when examining the association between CS and offspring's overweight/obesity, few studies have distinguished emergency CS from ECS, and the findings are conflicting. A Singaporean study (12) found that ECS was significantly associated with the risk of overweight in infants, whereas Masukume G et al indicated a statistically significant association between ECS and obesity in children at 24 months of age, but the association was not observed at 54 months of age (13). More importantly, there were no studies that intensively looked into the respective effect of ECS with or without medical indications on children's overweight/obesity. ECS without medical indications, without a doubt, obtains the great significance of preventability and intervention.

It has been well known that body mass index (BMI) fluctuates early in life (14). Tracking trajectory patterns over time during child development no longer focuses only on BMI at one point in time and can account for dynamic changes in size (15, 16). Compared to qualitative definition on overweight/obesity, BMI trajectories may better reflect the underlying dynamic patterns of individual's physical development over the life course. Adiposity rebound (AR) is defined as a second rise in BMI, usually occurring in early childhood. Studies have shown that early adiposity rebound (EAR) increased the risk of childhood obesity and is strongly associated with metabolic syndrome (17, 18) and cardiovascular diseases (19, 20). A recent meta-analysis found an increasing trend in the prevalence of EAR, suggesting that the age of AR in humans may be advancing compared to the past (21). AR, which relies on BMI trajectories, may be an early sensitive indicator of child physical growth and development.

The association between ECS and child growth trajectory, particularly with AR, has not been reported previously. Based on a large-sample birth cohort, this study aims to investigate the association between ECS and childhood BMI trajectories and early AR.

## Methods

### Study population

The participants were from the Ma'anshan-Anhui Birth Cohort (MABC) in China. Pregnant women were consecutively recruited from antenatal clinics of the Maternal and Child Health Care Center in Ma'anshan, Anhui Province, from May 2013 to September 2014 by trained investigators. The center contains about eighty percent of all pregnant women in Ma'anshan city every year. The inclusion criteria for women were (1) within 14 weeks of pregnancy; (2) planned to have antenatal checkups and childbirth in the hospital; (3) being able to understand and complete the questionnaires; and (4) willing to participate in follow-ups during childhood. A total of 3474 eligible women were recruited. After excluding 39 twins and 162 adverse pregnancy outcomes, 3273 women with live singleton births were obtained.

The study was approved by the ethical committee of Anhui Medical University (20131401). Written informed consents were obtained from all participants.

### Delivery mode

Data on delivery mode was extracted from the obstetrical records. Delivery mode was classified as CS and vaginal delivery (VD), thereafter CS was classified as ECS when it was performed before labor and emergency CS when it was performed after an attempt of vaginal delivery. Then ECS was further categorized as ECS with medical indicators and ECS without indicators.

### Assessment of children's BMI

Children's BMI was calculated as body weight (in kilograms) divided by body length or body height (in meters squared). Birth weight and birth length were extracted from the obstetrical records during childbirth. Children's body weight and length/height were measured by trained child healthcare providers. The infant's weight was measured using a pan-type lever scale, accurate to 0.01 kg. The toddler's weight was measured using a seated lever scale, accurate to 0.05 kg. The infant's length is measured by using a standard measuring bed, accurate to 0.1 cm. And the child's height and weight were measured using a mechanical child height and weight scale (Model: RRZ-50-RP) with an accurate reading of 0.1 kg for weight and 0.1 cm for height.

Children's body length/height and weight were repeatedly measured at 42 postnatal days, and 3, 6, 9, 12, 18, 24, 30, 36, 42, 48, 54, 60, 66, 72 months after birth. The raw BMI values in each age group were used for trajectory modeling.

### Covariates

Confounding variables were identified by directed acyclic graph (DAG) (22) and included maternal age, education level, place of residence, monthly household income per capita, smoking status, parity, pre-pregnancy BMI, weight gain during pregnancy, gestational diabetes mellitus (GDM) and hypertensive disorders in pregnancy (HDP).

Data on maternal age, education level, place of residence, family income, parity and smoking status were collected during the women's first antenatal visit by questionnaire survey. In this visit, women's body weight and height were measured, and the weight was regarded as pre-pregnancy body weight. Thus pre-pregnancy BMI was calculated from weight (kg) divided by the square of height (m^2^). Based on the guideline of Cooperative Meta-analysis Group of Working Group on Obesity in China (WGOC), BMI < 18.5 kg/m^2^ was defined as underweight, 18.5–23.9 kg/m^2^ as normal weight,24-27.9 kg/m^2^ as overweight and ≥28 kg/m^2^ for obesity for Chinese adults (23). Gestational weight gain was calculated by weight recorded shortly before childbirth subtracting the weight before pregnancy. GDM and HDP were abstracted from obstetrical notes.

Children's birth weight, gestational age, and exclusive breastfeeding were used in sensitivity analyses. Data on gestational age and birth weight were abstracted from obstetrical notes. A newborn with a birth weight below the 10th percentile for gestational age was defined as small for gestational age (SGA), between the 10th and 90th percentile for gestational age was defined as appropriate for gestational age (AGA) and above the 90th percentile for gestational age was defined as large for gestational age (LGA).

Exclusive breastfeeding was extracted from children's follow-up questionnaire survey. In this study, taking into account the actual situation of the Chinese population, exclusive breastfeeding was defined as infants who were not given any liquid or solid food, including water juice or vitamins other than breast milk.

### Statistical analysis

#### Trajectory modeling

##### Sample selection

Typically, trajectory fitting requires a minimum of 3 BMI measurements. This is because fewer measurements can limit the modeling capabilities and, therefore, the type and number of trajectories that can be generated (16, 24, 25). To ensure the quality of the trajectory fitting, among the 3273 newborns included in the MABC, 233 children were excluded because BMI data was < 3 times before 18 months. As data for AR was requested more stringently after 18 months, 651 children were excluded because of < 2 BMI measurements between 18 months and 3 years and < 3 measurements between 3 and 6 years, leading to a sample of 2389 children.

**Figure 1:**
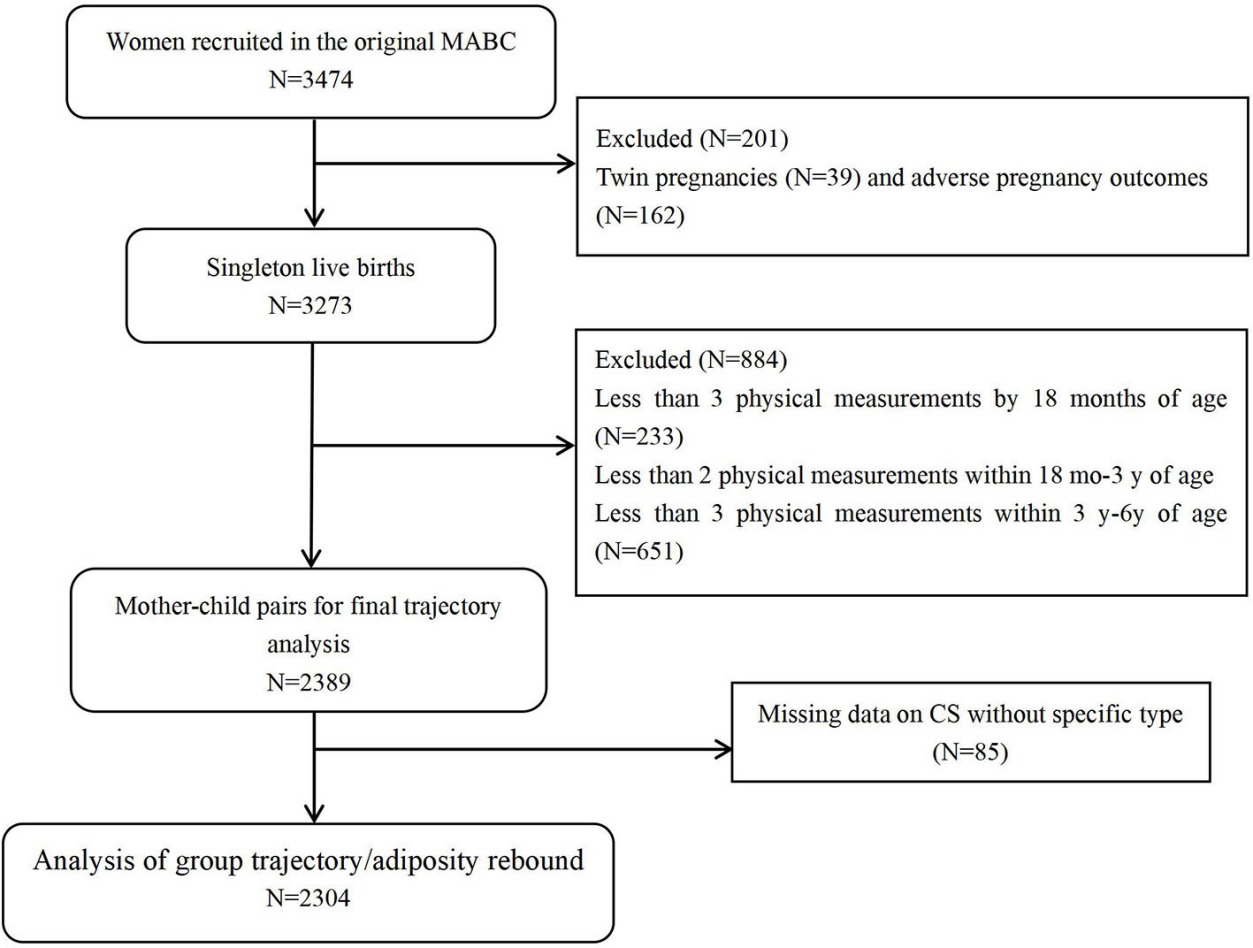
Flowchart of study participants.

Furthermore, infants with missing data on CS cause (*n* = 85) were further excluded, resulting in a complete sample of 2304 mother-infant pairs (Figure 1). Trajectories fitting was performed by using Stata 15.1 software.

#### Group-based trajectory

A group-based trajectory model (GBTM) was used to identify distinctive patterns of children's growth observed from birth to 6 years. GBTM is a latent class growth model designed to identify groups of trajectories, i.e., groups of individuals that follow similar trajectories over time. In GBTM, the optimal number of trajectory groups with different trajectory patterns was determined by the Bayesian information criterion (BIC). The group with the highest BIC value and polynomial are basically chosen in order. Usually, objective criteria are used for modeling, but subjective judgment is also essential (26). The number of groups or the polynomial order can be specified with professional knowledge. The final model should be simple while adequately presenting the data's characteristics (26) Therefore, the GBTM was used to fit the trajectories of children aged 0–6 years. Due to the large number of age groups involved and the large temporal variability of BMI in the early stages of child development, high-order terms (4 order terms) were used to fit the trajectories. The optimal model was selected based on the model parameters. For the category fitting, the number of categories increased from 2, based on the BIC of each fitted model, until a suitable trajectory category and a suitable combination of higher terms were selected.

#### Individual-specific trajectories

Individual-specific BMI trajectories were fitted from month 1 to month 72 of age by using a fractional polynomial mixed-effects model. Specific steps have been described in detail in wen's study (27). Briefly, children's BMI trajectory was firstly modeled as a function of age using a fractional polynomial approach. The expected value of BMI was modeled as $E(BMI)={\text{b}}_{0}+{\sum_{j=1}^{m}b_{j}Age^{P_{j}}}^{\text{​}}$, where m was the degree of the model, and powers P_j_ were selected from a fixed set of 8 candidate values, including −2, −1, −0.5, 0 or log, 0.5, 1, 2, and 3. Since the trajectory of children's BMI firstly rises, and falls after 1 year of age and then rises again after 4–5years of age, we set the minimum model degree m = 3. There are 219 candidate models, including 56 models of 3rd-degree, 70 models of 4th-degree, 56 models of 5th-degree, 28 models of 6th-degree, 8 models of 7th-degree, and 1 model of 8th-degree. Afterward, BMI trajectories were fitted using mixed-effects models. The models specified fixed effects for each fractional polynomial term, which reflected the overall average trend, and random effects for each term for each child as well, which had modeled individual differences. Then the trajectory characteristics of the candidate models were combined with the BIC value to select the best model. The smaller the BIC value was, the better the model fit would be. Estimates for AR were obtained from the BMI trajectories for each child. In the present study, age at AR before 54 months was defined as early AR. According to the estimated AR for each child, therefore, children were divided into early AR and non-early AR.

#### Association between ECS and children's growth trajectories and AR

IBM SPSS Statistics 23.0 was used to analyze the association between ECS and children's growth trajectories and AR. Multiple logistic regression models were adopted and stratifying by children's sex. For the association between ECS and children's growth trajectories, trajectory 2 was regarded as the reference. The independent variables were divided into ECS with medical indicators, ECS without indicators, emergency CS and VD, with vaginal delivery being as reference.

For the association between ECS and AR, non-early AR was regarded as the reference in the dependent variable. *P*-value of < 0.05 was considered significant.

We had performed two sensitivity analyses: First, there is an interaction between gestational week-birth weight and mode of delivery, and SGA is associated with catch-up growth leading to overweight/obesity (28). Thus, gestational week-birth weight may be a confounder and mediator in the association between cesarean delivery and child physical development. In a second sensitivity analysis, breastfed children exhibit lower trajectories of BMI-Z scores (29), and early access to complementary foods is associated with obesity in later childhood (30). Thus, exclusive breastfeeding is not a strict confounder but a precision variable.

## Results

### Basic characteristics of participants

Among 2304 children, 1216 (52.8%) were born vaginally and 1088 (47.2%) were born by CS, including 61 (5.6%) emergency CS, 441 (40.5%) ECS with medical indications, and 586 (53.9%) ECS without medical indications. A higher rate of exclusive breastfeeding in the first 4 months was found in girls than in boys (48.5% vs. 41.4%), and a higher proportion of girls were SGA (11.2% v.8.5%) and a higher proportion of boys were LGA (17.8% vs. 13.5%) (Table 1)

**Table 1:** Basic characteristics of participants (*n* = 2304).

**Variables**	**Total**	**Boys**	**Girls**
**Demographic Characteristics**	2304	1199	1105
Maternal age (Mean ± SD)	26.7 ± 3.7	26.8 ± 3.7	26.7 ± 3.6
Education level [*n* (%)]			
≤ 9	441 (19.1)	234 (19.5)	207 (18.7)
9–12	510 (22.1)	255 (21.3)	255 (23.1)
12–16	727 (31.6)	388 (32.4)	339 (30.7)
≥16	626 (27.2)	322 (26.9)	304 (27.5)
Place of residence [*n* (%)]			
Urban areas	2121 (92.1)	1106 (92.2)	1015 (91.9)
Rural areas	183 (7.9)	93 (7.8)	90 (8.1)
Monthly household income per capita [yuan, *n* (%)]			
< 2500	626 (27.2)	335 (27.9)	291 (26.3)
2500–4000	961 (41.7)	495 (41.3)	466 (42.2)
>4000	717 (31.1)	369 (30.8)	348 (31.5)
**Maternal characteristics**			
Smoking [*n* (%)]			
Yes	96 (4.2)	52(4.3)	44(4.0)
No	2208 (95.8)	1147(95.7)	1061(96.0)
Pre-pregnancy BMI^*^ (kg/m^2^)[*n* (%)]			
< 18.5	517 (22.8)	267 (22.7)	250 (22.9)
18.5–23.99	1505 (66.4)	786 (66.7)	719 (66.0)
24–27.99	200 (8.8)	100 (8.5)	100 (9.2)
>28	46 (2.0)	25 (2.1)	21 (1.9)
Weight gain during pregnancy^*^ (kg) (Mean ± SD)	17.8 ± 5.0	17.7 ± 5.0	17.8 ± 5.0
Parity [*n* (%)]			
Primiparity	2049 (88.9)	1061 (88.5)	988 (89.4)
Multiparity	255 (11.1)	138 (11.5)	117 (10.6)
GDM [*n* (%)]			
Yes	303 (13.2)	153 (12.8)	150 (13.6)
No	2001 (86.8)	1046 (87.2)	955 (86.4)
HDCP [*n* (%)]			
Yes	136 (5.9)	67 (5.6)	69 (6.2)
No	2168 (94.1)	1132 (94.4)	1036 (93.8)
Mode of delivery [*n* (%)]			
ECS with medical indication	441 (19.1)	218 (18.2)	223 (20.2)
ECS without medical indication	586 (25.4)	299 (24.9)	287 (26.0)
Emergency CS	61 (2.6)	36 (3.0)	25 (2.3)
VD	1216 (52.8)	646 (53.9)	570 (51.6)
**Children's characteristics**			
Gestational age(weeks) (Mean ± SD)	39.5 ± 1.3	39.4 ± 1.3	39.5 ± 1.3
Birth weight by gestational age [*n* (%)]			
SGA	226 (9.8)	102 (8.5)	124 (11.2)
LGA	362 (15.7)	213 (17.8)	149 (13.5)
AGA	1716 (74.5)	884 (73.7)	832 (75.3)
Exclusive breastfeeding at 4 months of age^*^ [*n* (%)]			
Yes	1015 (44.8)	490 (41.4)	525 (48.5)
No	1252 (55.2)	695 (58.6)	557 (51.5)

The ^*^ symbol indicate the variables with missing values.

### Grouping of BMI trajectories

**Figure 2:**
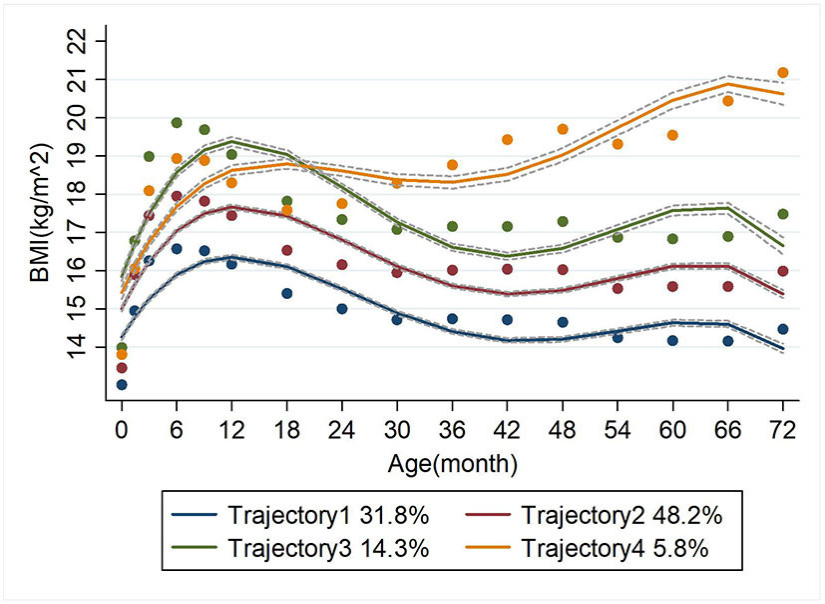
BMJ trajectory fitting for children aged 0–6 year.

Four BMI trajectory groups were identified. Trajectory 1 was the 'low level' group, representing a consistently low level of BMI with increasing age, accounting for 31.8%. Trajectory 2 was the 'middle level' group, representing a stable medium BMI with age, accounting for 48.2%. Trajectory 3 was the “high level” group, representing a consistently high level of BMI with age and accounted for 14.3%. And trajectory 4 was the 'accelerated growth' group accounting for 5.8% (Figure 2).

### Distribution of children's BMI trajectories and EAR under different delivery modes

As shown in Table 2, in boys, boys born *via* ECS without medical indicators and girls born by emergency CS had the most proportion of the “high level” BMI trajectory. In both boys and girls, those born *via* ECS with medical indications were more likely to have the 'accelerated growth' trajectory.

**Table 2:** Distribution of children's BMI trajectories and EAR with different delivery modes [*n* (%)].

**Mode of delivery**	**Boys**					**Girls**				
	**Traj 1**	**Traj 2**	**Traj 3**	**Traj 4**	**EAR**	**Traj 1**	**Traj 2**	**Traj 3**	**Traj 4**	**EAR**
**ECS with medical indicators**	44 20.2%	111 50.9%	42 19.3%	21 9.6%	98 45%	81 36.3%	99 44.4%	28 12.6%	15 6.7%	99 44.4%
**ECS without medical indicators**	81 27.1%	143 47.8%	61 20.4%	14 4.7%	119 39.8%	106 36.9%	134 46.7%	35 12.2%	12 4.2%	148 51.6%
**Emergency** **Cs**	6 16.7%	21 58.3%	6 16.7%	3 8.3%	19 52.8%	6 24.0%	13 52%	5 20%	1 4%	13 52%
**Vaginal delivery**	183 28.3%	318 49.2%	98 15.2%	47 7.3%	258 39.9%	221 38.8%	284 49.8%	45 7.9%	20 3.5%	233 40.9%

Basically, children born by CS had higher rate of EAR than those born by VD. The prevalence of EAR was higher in children born by emergency CS in both boys and girls.

### Association between ECS and children's BMI trajectories in boys and girls

As shown in Table 3, after adjusting for potential confounders, it was found that, among girls, ECS with medical indications births significantly associated with the “high level” trajectory (trajectory 3) (OR = 1.776, 95% CI: 1.010–3.123). Boys born by ECS without medical indication were less likely to have an “accelerated growth” trajectory (trajectory 4) (OR = 0.484, 95%CI: 0.244–0959).

**Table 3:** Association between ECS and children's growth trajectories in boys and girls [OR (95%CI)].

**Mode of delivery**	**sex**	**Model 1**			**Model 2**		
		**Traj1**	**Traj3**	**Traj4**	**Traj1**	**Traj3**	**Traj4**
**ECS with medical indicators**	**Boys**	0.689(0.465–1.021)	1.228(0.806–1.971)	1.280(0.733–2.236)	0.722(0.473–1.102)	1.084(0.695–1.690)	0.793(0.423–1.485)
	**Girls**	1.051(0.747–1.481)	**1.785 (1.057–3.015)**	**2.152 (1.060–4.365)**	1.224(0.848–1.766)	**1.776 (1.010–3.123)**	1.982(0.920–4.270)
**ECS without medical indicators**	**Boys**	0.984(0.709–1.366)	1.384(0.951–2.015)	0.662(0.353–1.242)	1.099(0.778–1.552)	1.293(0.874–1.912)	**0.484 (0.244–0.959)**
	**Girls**	1.017(0.746–1.385)	**1.648 (1.013–2.683)**	1.272(0.604–2.677)	1.169(0.844–1.620)	1.586(0.947–2.656)	1.231(0.564–2.685)
**Emergency** **CS**	**Boys**	0.496(0.709–1.366)	0.927(0.364–2.362)	0.967(0.278–3.367)	0.403(0.134–1.218)	0.786(0.303–2.036)	0.837(0.229–3.061)
	**Girls**	0.593(0.222–1.585)	2.427(0.826–7.135)	1.092(0.136–8.777)	0.576(0.199–1.668)	2.878 (0.927–8.931)	1.227 (0.144–10.423)

For delivery modes, vaginal delivery was the reference group. For BMI trajectories, Traj2 was regarded as the reference group.

Model 1, Crude model; Model 2, Adjusted for maternal age, maternal education level, residence, family income, smoking, pre-pregnancy BMI, weight gain during pregnancy, parity and pregnancy complications.

Sensitivity analysis did not change the main findings (Supplementary Tables S1–S2)

### Association between ECS and children's EAR in boys and girls

After adjusting for potential confounders, in girls, ECS without medical indicators was associated with the occurrence of the EAR (OR = 1.517 95%CI = 1.123-2.050). No significant association was observed between ECS and boys' prevalence of EAR (OR=.0994 95%CI=0.751-1.316 for ECS with medical indicators; OR = 0.857 95%CI=0.639-1.149 for ECS without medical indicators) (Table 4). Sensitivity analysis did not change the main findings fundamentally (Supplementary Tables S3–S4).

**Table 4:** Association between ECS and children's EAR in boys and girls [OR (95%CI)].

**Mode of delivery**	**Model 1**		**Model 2**	
	**boys**	**girls**	**boys**	**girls**
**ECS with medical indicators**	1.228 (0.901–1.674)	1.155 (0.845–1.578)	1.047 (0.751–1.460)	1.124 (0.803–1.573)
**ECS without medical indicators**	0.994 (0.751–1.316)	**1.540** **(1.158–2.049)**	0.857 (0.639–1.149)	**1.517** **(1.123–2.050)**
**Emergency** **Cs**	1.681 (0.857–3.295)	1.567 (0.703–3.495)	1.561 (0.764–3.191)	1.490 (0.611–3.248)

For delivery modes, vaginal delivery was the reference group. For AR, Not EAR was regarded as the reference group.

Model 1: Crude model; Model 2: Adjusted for maternal age, maternal education level, residence, family income, smoking, pre-pregnancy BMI, weight gain during pregnancy, parity and pregnancy complications.

## Discussion

In the current study, ECS was found to be associated with girl's BMI trajectory and timing of AR. In detail, ECS with medical indications was associated with “high level” BMI trajectory, and ECS without medical indication was related with girls' early AR.

Previous studies have reported the association between CS and children's BMI trajectories. Begum et al. (31)showed that the pooled OR of “high level” trajectory in offspring delivered by CS was 1.72 (95% CI: 1.28–2.31) compared with those born *via* VD. Zhang et al. (32) found that children delivered by CS (without distinguishing ECS and non-ECS) more possibly had a “medium to high increase” trajectory from 3 to 60 months of age and a “low to high increase” trajectory from 3 to 60 months of age. Similar to previous studies, we found an increased possibility of “high level” BMI trajectory in children born *via* ECS with medical indicators. “High level” trajectories are reported to have predictive values for obesity in later life (15). We did not find any association of emergency CS on children's BMI trajectories. Emergency CS is a procedure preceded by labor, where the membrane usually ruptures, resulting in exposure of the infant to vaginal microbiota. ECS, however, is performed before the onset of labor. A lack of infants' exposure to these microbes at birth may increase the risk of obesity later in life. Studies have found that CS lead to lower gut microbial diversity in children (33), with few species of the Bacteroides (34), a kind of beneficial bacteria. Decrease in the Bacteroides may increase the risk of obesity (35). Stress hormones triggered by different modes of delivery may also play a role. A normal increase in the stress hormone, cortisol, during childbirth facilitates the adaptation of the newborn to the extra-uterine environment (36). Studies have shown that infants born through ECS have the lowest stress response, lower response in emergency CS, and higher response in VD (37, 38). Reduced fetal cortisol levels in the ECS may lead to metabolic disorders at birth and future obesity (39).

In the current study, particularly, we found that girls born *via* ECS without medical indications were more possibly to have early AR. It is worth noting that ECS with medical indications is an effective means of saving women and children's life. It is necessary, and must be performed even if it would have certain impact on children's later health. ECS without medical indications is a non-essential procedure and is modifiable through informed choice by the health providers and health users. This is why we need to examine the respective effect of ECS with and without medical indications on maternal and children's health. Studies have proven that childhood early AR serves as an early predictor of obesity in later life (14, 17). Therefore, understanding the determinants of early AR, especially the modifiable factors, may improve the overall effectiveness of childhood obesity prevention. Due to ethical considerations, it is unfeasible to conduct randomized controlled trials to identify the impact of CS on maternal and infant health. In this study, findings of the association between ECS without medical indications and the risk of early AR and possible later onset of obesity will provide additional evidence-based advice to clinicians and women in their informed choice of delivery mode. It will also help to decrease the waste of medical resources for unnecessary CS and achieve much earlier prevention and control of childhood obesity.

The findings that ECS related with high level BMI trajectory and early AR were observed only in girls. This may be related to boys' and girls' physiology. Girls are genetically determined to be more prone to fat accumulation and obesity than boys (40), and have higher body fat than boys beginning in the second trimester of gestation and extending throughout life (41). The animal model also found that mice with two X chromosomes gained almost twice as much fat as mice with X and Y chromosomes (42).

Our study has some strengths. Firstly, to our knowledge, this is the first time that ECS has been subdivided into ECS with medical indicators and ECS without medical indicators in examining the association of ECS on children's physical development. As has not been previously reported, we found ECS without medical indication related with girls' early AR. ECS without medical indicators may violate eugenic principles and adversely affect offspring's physical development, and this type of delivery can exactly be avoided by rational informed consent toward women. In the second, there were exceedingly frequent follow-ups with children. Follow-up visits were conducted every 3 months until 1 year of age and every 6 months after 1 year of age. To date, we have collected children's complete data from 15 follow-up visits. This would permit a more accurate fitting for children's growth trajectory (15). In the third, based on a prospective birth cohort, information on exposure, outcome and confounders could be accurately collected, which decreased the recall bias. Meanwhile, precision variables were considered to effectively enhance the precision of the findings.

Some limitations must be acknowledged. Other postnatal factors, such as children's activities, sleep, diet also affect their physical development. Low activity levels and short sleep duration are reported to be associated with high BMI in pre-adolescent children (43, 44). These factors, however, had not been addressed in our study. Furthermore, paternal genetic factors may play vital roles in influencing children's physical development. For instance, paternal overweight and obesity and their potential inheritance have been shown to be a risk factor for children's high BMI (45). There was still a lack of father's anthropometric indicators in the current study.

In conclusion, in this population-based longitudinal study, ECS with medical indication was found to be associated with “high level” BMI trajectory, and ECS without medical indication was found to be associated with early AR in girls.

## Data availability statement

The datasets generated and/or analyzed during the current study are not publicly available but are available from the corresponding author on reasonable request.

## Ethics statement

The studies involving human participants were reviewed and approved by Ethical Committee of Anhui Medical University (20131401). Written informed consent to participate in this study was provided by the participants' legal guardian/next of kin.

## Author contributions

SZ collated, analyzed the data, and was the lead author of the first draft. JZ and XT were involved in the data analysis. MY was involved in the cohort data collection. FZ was involved in the literature collection. FT was the cohort leader. KH was the cohort site leader, reviewed, revised the first draft, supervised, and led the study. All authors contributed to the article and approved the submitted version.

## Funding

National Natural Science Foundation of China (81872630), The University Synergy Innovation Program of Anhui Province (GXXT-2020-067), Sci-tech Basic Resources Research Program of China (2017FY101107), the Non-profit Central Research Institute Fund of Chinese Academy of Medical Sciences (2019PT310002) and Research Fund of Anhui Institute of translational medicine (ZHYX2020A001).

## Conflict of interest

The authors declare that the research was conducted in the absence of any commercial or financial relationships that could be construed as a potential conflict of interest.

## Publisher's note

All claims expressed in this article are solely those of the authors and do not necessarily represent those of their affiliated organizations, or those of the publisher, the editors and the reviewers. Any product that may be evaluated in this article, or claim that may be made by its manufacturer, is not guaranteed or endorsed by the publisher.

## Abbreviations

CS: Cesarean Section
ECS: Elective Cesarean Section
VD: Vaginal Delivery
AR: Adiposity Rebound
EAR: Early adiposity rebound
BMI: Body Mass Index
MABC: Ma'anshan-Anhui Birth Cohort
GDM: G estational Diabetes Mellitus
HDP: Hypertensive Disorders in Pregnancy
SGA: Small for Gestational Age
AGA: Appropriate for Gestational Age
LGA: Large for Gestational Age
GBTM: Group-Based Trajectory Model
BIC: Bayesian information criterion.
