# Supplementary material for: Sex-specific association between elective cesarean section and growth trajectories in preschool children: A prospective birth cohort study
Source: Front Public Health. 2022 Sep 20;10:985851. doi: 10.3389/fpubh.2022.985851 (PMC9530938; doi:10.3389/fpubh.2022.985851)
Supplement: Supplementary file 1 [file Data_Sheet_1.pdf]

S-Table 1 Association between ECS and children's growth trajectories in boys and girls **[OR (95%CI)]**

**Mode of delivery**	**Sex**	**BMI trajectory**		
		**Traj1**	**Traj3**	**Traj4**
ECS with medical indicators	Boys	0.721 (0.472-1.104)	1.070 (0.681-1.680)	0.828 (0.437-1.567)
	girls	1.191 (0.821-1.726)	1.786 (1.008-3.165)	1.864 (0.841-4.129)
ECS without medical indicators	Boys	1.069 (0.751-1.520)	1.214 (0.810-1.821)	0.521 (0.259-1.048)
	girls	1.180 (0.848-1.643)	1.633 (0.963-2.768))	1.250 (0.552-2.832)
Emergency Cs	Boys	0.392 (0.130-1.186)	0.767 (0.295-1.993)	0.874 (0.236-3.235)
	girls	0.603 (0.206-1.769)	2.916 (0.917-9.274)	1.291 (0.150-11.133)

For delivery modes, vaginal delivery was the reference group. For BMI trajectories, Traj2 was regarded as the reference group.

On the basis of model 2 adjusted for gestational age.

S-Table 2 Association between ECS and children's growth trajectories in boys and girls **[OR (95%CI)]**

**Mode of delivery**	**Sex**	**BMI trajectory**		
		**Traj1**	**Traj3**	**Traj4**
ECS with medical indicators	Boys	0.721 (0.470-1.107)	1.106 (0.707-1.728)	0.804 (0.427-1.515)
	girls	1.204 (0.832-1.741)	1.785 (1.010-3.158)	1.941 (0.899-4.193)
ECS without medical indicators	Boys	1.117 (0.788-1.583)	1.321 (0.892-1.957)	0.495 (0.248-0.990)
	girls	1.144 (0.823-1.591)	1.647 (0.976-2.779)	1.096 (0.491-2.447)
Emergency Cs	Boys	0.387 (0.128-1.172)	0.792 (0.305-2.053)	0.799 (0.217-2.948)
	girls	0.565 (0.195-1.638)	3.074 (0.987-9.573)	1.133 (0.132-9.711)

For delivery modes, vaginal delivery was the reference group. For BMI trajectories, Traj2 was regarded as the reference group.

On the basis of model 2 adjusted for exclusive breastfeeding at the first 4 months.

S-Table 3 Association between ECS and children's EAR in boys and girls **[OR (95%CI)]**

**Mode of delivery**	**boys**	**girls**
**ECS with medical indicators**	1.075 (0.768-1.504)	1.142 (0.813-1.604)
**ECS without medical indicators**	0.868 (0.647-1.166)	1.539 (1.138-2.081)
**Emergency Cs**	1.642 (0.800-3.370)	1.468 (0.635-3.393)

For delivery modes, vaginal delivery was the reference group. For BMI trajectories, Traj2 was regarded as the reference group.

On the basis of model 2 adjusted for birth weight by gestational age.

Values highlighted in yellow are statistically significant.

S-Table 4 Association between ECS and children's EAR in boys and girls **[OR (95%CI)]**

**Mode of delivery**	**boys**	**girls**
**ECS with medical indicators**	1.074 (0.768-1.501)	1.134 (0.809-1.591)
**ECS without medical indicators**	0.872 (0.649-1.172)	1.517 (1.118-2.058)
**Emergency Cs**	1.606 (0.785-3.287)	1.423 (0.617-3.282)

For delivery modes, vaginal delivery was the reference group. For BMI trajectories, Traj2 was regarded as the reference group.

On the basis of model 2 adjusted for exclusive breastfeeding at the first 4 months.

Values highlighted in yellow are statistically significant.
